# Stroke risk perception among participants of a stroke awareness campaign

**DOI:** 10.1186/1471-2458-7-39

**Published:** 2007-03-20

**Authors:** Klaus Kraywinkel, Jan Heidrich, Peter U Heuschmann, Markus Wagner, Klaus Berger

**Affiliations:** 1 Institute of Epidemiology and Social Medicine, University of Muenster, Germany; 2 German Stroke Foundation, Guetersloh, Germany

## Abstract

**Background:**

Subjective risk factor perception is an important component of the motivation to change unhealthy life styles. While prior studies assessed cardiovascular risk factor knowledge, little is known about determinants of the individual perception of stroke risk.

**Methods:**

Survey by mailed questionnaire among 1483 participants of a prior public stroke campaign in Germany. Participants had been informed about their individual stroke risk based on the Framingham stroke risk score. Stroke risk factor knowledge, perception of lifetime stroke risk and risk factor status were included in the questionnaire, and the determinants of good risk factor knowledge and high stroke risk perception were identified using logistic regression models.

**Results:**

Overall stroke risk factor knowledge was good with 67–96% of the participants recognizing established risk factors. The two exceptions were diabetes (recognized by 49%) and myocardial infarction (57%). Knowledge of a specific factor was superior among those affected by it. 13% of all participants considered themselves of having a high stroke risk, 55% indicated a moderate risk. All major risk factors contributed significantly to the perception of being at high stroke risk, but the effects of age, sex and education were non-significant. Poor self-rated health was additionally associated with high individual stroke risk perception.

**Conclusion:**

Stroke risk factor knowledge was high in this study. The self perception of an increased stroke risk was associated with established risk factors as well as low perception of general health.

## Background

The individual perception of health risks is an accepted key issue when goals of primary and secondary prevention are defined. Common theories on health behaviour, such as the Health Belief Model [1] or the Protection Motivation Theory [2], support the importance of risk perception, also called 'perceived susceptibility', for health education and preventive medicine. An underestimation of personal risk could reduce the motivation for a change in behavior- dependent risk factors and could decrease compliance with medical prevention strategies. Especially for cardiovascular diseases, for which primary prevention has large potential benefits [3], adequate risk perception is an important step for the change of risk related lifestyles.

There have been a number of studies addressing the general perception of health risks and how inadequate risk perception could be moderated [4-6]. Other studies have examined perception in individuals presumed to have an overall high disease risk, e.g. smokers [7-9], or evaluated risk perception of specific diseases like coronary heart disease or heart attack [10,11]. For stroke, one of the leading causes of death and disability worldwide, studies about knowledge of warning signs and risk factors have been conducted in several countries involving different populations [12-19], but little is known about factors that contribute to the perception of stroke risk.

The aim of this study was to examine the influence of existing risk factors on an individual's perception of stroke risk.

## Methods

This study included participants of a stroke awareness campaign conducted by the German Stroke Foundation in 2003. The initial stroke campaign was published by advertisements in different German TV stations, newspapers and magazines and on a website. Participation was possible by completing a questionnaire either in paper form or via the internet, the latter approach was used by nearly half of the participants (48.7%). In this questionnaire individual risks for stroke and other vascular diseases were assessed through the Framingham Risk Score [20] using self-reported information about risk factors and comorbidities. Using traffic light symbols, participants received a written feedback if their stroke risk was normal ("green"), elevated (two to four-fold risk = "yellow") or clearly increased (more than four-fold risk = "red"), compared to individuals of the same age and gender. They were also informed about their individual risk factors and about ways to modify them.

In 2004 we recontacted 3066 former campaign participants for a follow-up survey by mailed questionnaire. Included were individuals without prior stroke who had given written consent to be recontacted for further surveys. The follow-up questionnaire included items on stroke knowledge, stroke risk perception and preferred sources of information about stroke and general health issues. In addition, motivation for lifestyle changes as well as various sociodemographic variables were assessed. Data were collected via mailing between April and July 2004, i.e. 3 to 12 months after participation in the initial stroke campaign.

The following definitions and methods were used in the assessment of risk factor knowledge and risk perception of stroke: fifteen possible risk factors for stroke were presented in the questionnaire. Participants were asked whether they regarded each item as a risk factor, possible answers were 'yes', 'no', or 'I don't know'. Four of these 15 suggested factors were no established risk factors for stroke (stress, shortness of sleep, liver diseases, myopia). The remaining eleven factors were a priori classified as accepted stroke risk factors (high alcohol intake, smoking, lack of exercise, unhealthy diet, hypertension, elevated blood lipids, myocardial infarction, diabetes, age, family history of stroke, prior stroke). Finally, the participants were asked to rate their lifetime risk of suffering a stroke as 'nonexistent', 'low', 'moderate' or 'high'. The chosen category was interpreted as the individual's perceived stroke risk. In addition, the questionnaire included various sociodemographic variables (age, sex, education). The individual's self-rated general health status was assessed using the first question of the Short-Form 36 (SF-36), a widely used quality of life questionnaire [21]. The respective answer was dichotomised for analysis ('moderate or poor' vs. 'good or excellent'). The participants' previously self-reported information collected during the initial stroke campaign was used for the classification of risk factors and comorbidities. The study was approved by the joint ethics committee of the Chamber of Physicians Westfalia and the University of Muenster (Ref. No. 50065321).

### Statistical analysis

A summary variable 'knowledge of stroke risk factors' was derived by calculating the percentage of correct answers to the 15 presented risk factors for each participant. Any positive identification of the 11 established risk factors as well as negative identification of the four others was considered as a correct answer, any 'don't know' answer was considered incorrect. Good and poor knowledge of stroke risk factors was subsequently defined according to the lower and upper tertiles of the distribution of correct answers. This resulted in cut-off points of < 65% (poor knowledge) and = 75% (good knowledge). The intermediate tertile (65–74%) was labelled moderate knowledge. History of myocardial infarction, atrial fibrillation and other heart diseases were summarized to one combined variable (heart disease). High education was defined as completion of at least the 12^th ^grade of school. With the exception of Body Mass Index (BMI < 25; 25–30, > 30 kg/m^2^) and smoking (current, ex, never) all risk factors were treated as dichotomized variables.

Chi-Square tests were performed to test for group differences in categorical variables. Logistic regression models were used to assess the impact of existing risk factors, sociodemographic variables and perceived stroke risk on risk factor knowledge. The dependent variable in these models was good risk factor knowledge versus moderate/bad knowledge. Odds ratios with 95% confidence intervals were calculated controlling for age and subsequent adjustment for gender, education, diabetes, heart disease, smoking, hypertension, obesity, hyperlipidemia, physical activity, familiar history of stroke or myocardial infarction and high risk perception. Logistic regression was also applied to determine independent predictors for stroke risk perception. In this analysis the perception of high stroke risk versus all other perceptions was the dependent variable.

When information on a risk factor was missing (< 10% for each variable), absence of the risk factor was assumed. In a sensitivity analysis, all models were computed in cases with complete information for all variables only (N = 1012). All computations were performed with SPSS 11.0 (SPSS Inc., Chicago, Illinois).

## Results

From the 3066 contacted individuals 1483 completed the questionnaire, yielding a response rate of 48.4%. Participants mean age was 47.0 years and table 1 summarizes their risk factors and comorbidities. Table 2 shows risk factor knowledge. The best known risk factors were previous stroke, hypertension and smoking, less well known were previous myocardial infarction and diabetes. Overall, 68,6 % of the questions about stroke risk factors were correctly answered. While 83,6% of the participants rated 'stress' as being a risk factor, shortness of sleep, liver diseases, and myopia were judged as risk factors only by 18.0%, 4.9% and 1.1%, respectively. For 34.1 % of the participants hypertension was the single most important risk factor, followed by smoking (17.6%). With the exception of low physical activity, people affected by a specific risk factor were more likely to recognize its importance as a risk factor for stroke. Physically active individuals more often rated low activity as a risk factor compared to inactive persons. This difference in perceived importance between affected and unaffected participants was significant for diabetes, hypertension, hyperlipidemia and family history of stroke or myocardial infarction and physical activity (table 2).

**Table 1:** Characteristics of the study population (n = 1483)

Variable ^a^	%
Age groups	
< 40 years	30.3
41–60 years	51.0
> 60 years	18.8
Female gender	43.2
High education (≥ 12 school years)	46.4
History of diabetes	4.2
History of hypertension	29.0
History of myocardial infarction	2.0
Current smoker	21.8
Hyperlipidemia	31.2
Family history of stroke or myocardial infarction	52.0
Low physical activity	43.8

^a ^All variables are self-reported

**Table 2:** Knowledge of specific stroke risk factors, stratified by own risk factor status

	Correctly identified risk factor			
		
Risk factor	All participants	Participants affected by this factor	Participants unaffected by this factor	p for difference between affected and uneffected
Precedent stroke	96,1%	-	96,1%	-
Hypertension	93,4%	96,6%	92,2%	0,002
Smoking	92,1%	93,1%^a^	90,6%	0,10
Hyperlipidemia	84,7%	93,0%	82,1%	< 0,001
Age	80,0%	80,3%^b^	79,8%^c^	0,89
Low physical activity	79,1%	75,8%	81,7%	0,01
Unhealthy diet ^d^	74,7%	-	-	-
Family history of stroke or myocardial infarction	74,2%	80,5%	67,9%	< 0,001
High alcohol intake ^d^	67,1%	-	-	-
Myocardial infarction	57,5%	67,9%	57,0%	0,29
Diabetes	49,3%	86,4%	47,9%	< 0,001

^a ^Current or ex-smokers

^b ^age ≥ 55 years

^c ^age < 55 years

^d ^Individual risk factor status not assessed

In multivariate analysis, female gender, higher education, history of diabetes and family history of stroke or myocardial infarction were independent predictors of good stroke risk factor knowledge. In contrast, existing heart disease and low physical activity were associated with a lower probability of good knowledge (table 3). When additionally included in the model, an elevated Framingham risk score was associated with a lower probability of good risk factor knowledge (OR 0.49, 95% CI 0.35, 0.69).

**Table 3:** Determinants of good stroke risk factor knowledge (highest tertile of risk factor knowledge compared to remainder)

Variable (self-reported)	age-adjusted model ^a ^OR (95% CI)	multivariable model^b ^OR (95% CI)
Diabetes	1.80 (1.07–3.05)	1.95 (1.14–3.35)
Heart disease ^d^	0.66 (0.44–0.99)	0.62 (0.40–0.95)
Smoking (current) (ex)	0.86 (0.63–1.17) 1.14 (0.89–1.46)	0.94 (0.68–1.29) 1.20 (0.93–1.55)
Hypertension	0.99 (0.76–1.28)	1.05 (0.80–1.38)
Obesity (BMI > 30) (BMI 25–30)	0.99 (0.71–1.37) 0.91 (0.71–1.16)	1.00 (0.77–1.30) 1.01 (0.72–1.41)
Hyperlipidemia	1.18 (0.92–1.51)	1.18 (0.91–1.52)
Low physical activity ^e^	0.76 (0.60–0.96)	0.76 (0.59–0.96)
Family history of stroke or myocardial infarction	1.41 (1.13–1.77)	1.40 (1.11–1.76)
Female gender	1.28 (1.02–1.60)	1.27 (1.00–1.62)
Higher education ^f^	1.33 (1.06–1.66)	1.36 (1.08–1.71)
Age (per 10 year increase)	1.04 (0.96–1.13)^c^	1.03 (0.93–1.14)

^a ^Odds ratios (OR) and 95% confidence intervals (95% CI) derived from logistic regression and adjusted for age

^b ^Additionally adjusted for all other reported variables in table 3

^c ^Unadjusted

^d ^History of myocardial infarction, atrial fibrillation or other heart diseases

^e ^Less than 2 hours a week

^f ^Completion of > = 12 grades of school

Of all study participants, 13% considered themselves of having a high lifetime risk for stroke, while 55% indicated a moderate and 32% either a low or no risk. Of the 299 participants with an elevated Framingham Risk Score (yellow traffic light symbol), 25.5% perceived a high, and 58.5% a moderate risk for stroke. Only 20 participants had received information about a strongly increased stroke risk (red light), of which 60% rated themselves as having a high risk and 35% having a moderate risk in the follow-up survey. In multivariate analysis, affection by specific risk factors had a significant impact on the perception of an increased stroke risk (table 4). Even moderate overweight (BMI 25–30) considerably increased the participants' judgement of their personal stroke risk. Poor perception of general health also strongly contributed to a perception of high stroke risk, but neither gender, nor education or age were significant predictors in the multivariate model. When additionally included in the model, an elevated Framingham risk score was also an independent predictor of high stroke risk perception (OR 2.52; 95% CI 1.73, 3.68). When the multivariate analysis was restricted to cases with complete information (n = 1012), the results remained largely unchanged. The only substantial changes in the magnitude of the odds ratios occurred for hyperlipidemia (1.54 to 1.93) and self perceived health (3.02 to 4.10).

**Table 4:** Determinants of high stroke risk perception

Variable (self-reported)	age-adjusted model ^a ^OR (95% CI)	multivariable model^b ^OR (95% CI)
Diabetes	3.99 (2.28–6.98)	2.98 (1.61–5.11)
Heart disease ^d^	2.83 (1.86–4.32)	1.79 (1.10–2.91)
Smoking (current) (ex)	1.63 (1.10–2.43) 1.09 (0.76–1.55)	1.81 (1.18–2.79) 1.01 (0.69–1.48)
Hypertension	2.48 (1.77–3.46)	1.65 (1.14–2.40)
Obesity (BMI > 30) (BMI 25–30)	2.93 (1.93–4.45) 2.14 (1.50–3.06)	2.11 (1.34–3.33) 2.22 (1.51–3.26)
Hyperlipidemia	1.88 (1.36–2.60)	1.54 (1.09–2.18)
Low physical activity ^e^	1.38 (1.00–1.89)	1.10 (0.78–1.54)
Family history of stroke or myocardial infarction	1.50 (1.10–2.05)	1.49 (1.07–2.09)
Female gender	1.04 (0.76–1.41)	1.23 (0.87–1.74)
Higher education^f^	0.61 (0.44–1.85)	0.78 (0.55–1.10)
Age (per 10 year increase)	1.12 (0.99–1.25) c	0.90 (0.78–1.04)
Poor general health perception	4.04 (2.82–5.79)	3.02 (2.03–4.48)

^a ^Odds ratios (OR) and 95% confidence intervals (95% CI) derived from logistic regression and adjusted for age

^b ^Additionally adjusted for all other reported variables in table 4

^c ^Unadjusted

^d ^History of myocardial infarction, atrial fibrillation or other heart diseases

^e ^Less than 2 hours a week

^f ^Completion of > = 12 grades of school

## Discussion

In this follow-up study of participants in a stroke awareness campaign the overall knowledge of important stroke risk factors 3 to 12 months after the initial campaign was generally high. In particular, participants with a specific risk factor were well informed about its significance. Good knowledge of risk factors was associated with history of diabetes, high physical activity, high education and female gender. In contrast, an increased stroke risk as measured by the Framingham Risk Score was inversely associated to good risk factor knowledge. One explanation for the latter finding is that a significant proportion of our study population may be described as the so called 'worried well' [22], i.e. individuals with a subjective perception of an increased stroke risk who actually do not have relevant risk factors. These 'worried well' may be better informed about general health issues. In contrast, individuals with increased 'objective' risks might be more interested in the consequences of those risk factors they are affected by. Physical active individuals more often indicated low activity as a risk factor. This observation seems plausible since those who do at least some exercise are likely to believe in a health benefit of their efforts. Stress was indicated as a risk factor by the majority of participants similar to several precedent studies [17,23,24]. Although self reported psychological stress was not found to be an independent risk factor for stroke in the Copenhagen City Heart Study [25], there are other findings that adaptive behavior in stressful situations may influence stroke risk [26], and that stress reduction could result in reduction of atherosclerosis [27] and mortality in hypertensives [28]. Although it might be debatable if stress could be considered a risk factor for stroke, it can be assumed that the concept of stress as a health hazard differs between medical professionals and the lay population.

Previous studies [29,30] have demonstrated that individualized feedback on cardiovascular risk factors have some effect on risk perception. In our study, only 27% of those who were informed about increased stroke risk before actually perceived a high stroke risk during the follow-up survey. There are several possible explanations for this low proportion. Changes in behavior dependent risk factors (e.g. smoking cessation) or newly started medical treatment (e.g. of hypertension) may have resulted in a more optimistic self perception of risks over time. Other participants may not have remembered their results. An evaluation of a different stroke campaign in 2001 showed that only 66% of those informed about high risk actually recalled their result correctly during a telephone interview 2 to 6 months later (German Stroke Foundation, unpublished observation). Finally, other studies demonstrated that when asked about perception of health risks most people tend to show an optimism bias [31,32] and rarely choose the extreme category with the 'maximum' risk.

Similar to prior studies on cardiovascular disease risks [33, 34], we found an increased perception of stroke risk in participants with subjective perception of poor general health. This relation was largely independent of reported stroke risk factors. One possible explanation for this finding is that people with lower general health perception are more likely to visit their physicians and therefore to be informed about the significance of their individual risk factors. In contrast, those who generally feel well might worry less about health risks. Furthermore, it is well known that self perceived health is a predictor for overall mortality, independent of established risk factors, which suggests an association with unmeasured risk factors [35]. The impact of moderate overweight on stroke risk perception was remarkably high in our study. Obesity is not included in the Framingham risk score and therefore had no effect on the feedback participants received during the initial campaign. While health implications of moderate overweight are still debated [36], its significance might have been overemphasized by the participants compared to that of other risk factors.

Our study has strengths and limitations. The study population is supposed to be highly selected with regard to an interest in health issues and, further, by the potential for response bias. A comparison to the German National Health survey 1997/98 [37] showed that men, individuals aged 41–60 years, people with higher education and non-smokers were overrepresented in our study while the proportions of participants with a history of diabetes, hypertension or myocardial infarction were comparable to those reported for the German population. Due to this selected study population our results cannot be generalized to the general population but more likely represent those who are especially interested in information about health issues. However, these are the usual clients of health actions and information campaigns in most countries and health care systems. Another limitation is the fact that all risk factors were self-reported which might have resulted in some misclassification of risk factor status. We used closed questions which facilitated participation since our questionnaire included several sections with different topics. We had successfully used these methods before [18] by adapting questions developed by Samsa et al. [8]. Closed questions might lead to a higher percentage of correct answer compared to open ended questions or semi structured interviews, used in several other studies before. Therefore, we did not directly compare our results to those of similar studies from other countries, and rather focused on the associations between existing risk factors and risk factor knowledge as well as risk perception. Among the strengths is the study design that enabled us to make use of the detailed baseline information assessed during the initial stroke campaign and to analyse the data according to baseline stroke risk status.

## Conclusion

In summary, good knowledge of stroke risk factors was found among participants 3 to 12 months after a public stroke awareness campaign. Existing risk factors contributed to the perception of an increased stroke risk as did poor self-perceived general health status, but we found substantial differences between subjective stroke risk perception and the results of the Framingham stroke risk score (even though detailed information about individual stroke risk had been given). Although our study population was highly selected, the results of our study may contribute to the understanding of stroke risk factor knowledge and in particular stroke risk perception among individuals with interest in health issues.

## Competing interests

The author(s) declare that they have no competing interests.

## Authors' contributions

KK performed the statistical analysis and drafted the manuscript, JH contributed to the acquisition and interpretation of the data and revising the manuscript, PUH participated in the design of the study and contributed to the interpretation of the data, MW contributed to the acquisition of the data and the critical revision of the manuscript, KB contributed to the conception and design of the study, the acquisition of data and helped to draft the manuscript. All authors read and approved the final manuscript.

## Pre-publication history

The pre-publication history for this paper can be accessed here:


